# Neuropsychological Profile in a Large Group of Heart Transplant Candidates

**DOI:** 10.1371/journal.pone.0028313

**Published:** 2011-12-13

**Authors:** Daniela Mapelli, Lara Bardi, Marco Mojoli, Biancarosa Volpe, Gino Gerosa, Piero Amodio, Luciano Daliento

**Affiliations:** 1 Department of General Psychology, University of Padua, Padua, Italy; 2 Interdepartmental Centre for Research on Modelling of Neuropsychological Disorders in Clinical Medicine-CIRMANMEC, University of Padua, Padua, Italy; 3 Department of Cardiological, Thoracic and Vascular Sciences, University of Padua, Padua, Italy; 4 Department of Clinical and Experimental Medicine, University of Padua, Padua, Italy; University of Bologna, Italy

## Abstract

**Background:**

Recent studies have reported that patients with end-stage heart disease can have cognitive deficits ranging from mild to severe. Little is known, however, about the relationship between cognitive performance, neurophysiological characteristics and relevant clinical and instrumental indexes for an extensive evaluation of patients with heart failure, such as: left ventricular ejection fraction (LVEF) and other haemodynamic measures, maximum oxygen uptake during cardiopulmonary exercise testing, comorbidities, major cardiovascular risk factors and disease duration. Our purpose was to outline the cognitive profiles of end-stage heart disease patients in order to identify the cognitive deficits that could compromise the quality of life and the therapeutic adherence in end-stage heart disease patients, and to identify the variables associated with an increased risk of cognitive deficits in these patients.

**Methods:**

207 patients with end-stage cardiac disease, candidates for heart transplant, were assessed by complete neuropsychological evaluation and by electroencephalographic recording with EEG spectral analysis.

**Results:**

Pathological scores in one or more of the cognitive tests were obtained by 86% of the patients, while 36% performed within the impaired range on five or more tests, indicating poor performance across a broad range of cognitive domains. The executive functions were the cognitive domain most impaired (70%). Poor performances were not related to the aetiology of heart disease, but rather to cerebral dysfunction secondary to haemodynamic impairment and to comorbidities.

**Conclusions:**

Severe heart failure induces significant neurophysiological and neuropsychological alterations, which may produce an impairment of cognitive functioning and possibly compromise the quality of life of patients and the therapeutic adherence.

## Introduction

Low cardiac output associated with heart failure often leads to end-organ damage including brain injury. In 1977, heart failure was proposed as a possible cause of cognitive dysfunction, introducing the term “cardiogenic dementia” [1]. However, only a limited number of clinical studies have systematically evaluated the cognitive profile of heart failure patients [2]. Furthermore, most of these studies only focused on elderly patient populations in which a high prevalence of cognitive deficits and a decrease in the quality of life were demonstrated in the case of severe heart failure [3]–[5]. There is also considerable evidence to show that cognitive impairments in geriatric patients with cardiovascular disease are due to cerebral hypoperfusion secondary to impaired cardiac function since the homeostatic mechanisms involved in maintaining haemodynamic regulation break down as people age [6], [7].

In contrast, relatively few studies have examined the neuropsychological performance in younger patients scheduled for cardiac transplantation, even though some early studies [8]–[10] revealed a high incidence of organic neurologic complications in cardiac transplant patients. Moreover, few of them investigated a large range of cognitive functions in a large sample of end-stage heart disease patients [11], [12]. The study by Putzke and colleagues provided descriptive neuropsychological data for a very large group of 760 patients, and certainly improved on previous studies that were limited by a relatively small sample size [13], [14]. Their results showed that the types of cardiac diagnosis and cardiac surgical history were not related to cognitive functioning. This finding increased the need to consider other clinical features and comorbidity factors that might be more closely related to cognitive impairment in end-stage disease patients [15]. Moreover, it has been previously observed that cerebrovascular complications associated with heart failure may lead to abnormalities in the brain structure [16], [17]. However, how brain abnormalities are associated with clinical factors and cognitive performance in end-stage heart disease patients is poorly understood. Studies of cognitive function in the context of heart transplantation are needed for several reasons. Patients must understand and participate in the decision-making process before surgery. Furthermore, cognitive functions can influence the ability to understand and to adhere to post-transplant treatment protocols. Neurological and/or neuropsychological morbidity before, during and after heart transplantation is not infrequent. Cognitive disorders could affect the basic activities of daily living, such as, for example, driving, financial planning, bill paying, shopping, meal preparation ect. Finally, studies on patients who need heart transplantation may provide data for a better understanding of the mechanisms underlying cognitive deficits in any severe heart disease population.

Basically, four important issues surrounding this population of patients need to be explored: [1] Is it possible to define a characteristic cognitive profile for this population? Are there some cognitive functions that are more impaired than others? Are those cognitive functions involved in adherence to therapy extensively compromised? [2] Is there any relationship between specific cognitive deficits, aetiology and the natural history of cardiopathy? [3] Are there any relationships between a single parameter of haemodynamic failure or any other clinical findings and severity or specificity of cognitive deficits? [4] How indexes of brain functioning (EEG) may be related to parameters of hemodynamic failure and cognitive performance in these patients?

We conducted a very complete neuropsychological and neurophysiological assessment in order to identify the particular profile of end-stage cardiac patients. Furthermore, we studied the executive functions in detail since that they are important not only for the quality of life but also for the management of therapy and medication adherence [18]–[20], as taking medicines consistently involves developing and implementing a plan of adherence and remembering to adhere to it. Medication adherence strongly depends on prospective memory and may also involve working memory, since the patient must keep the intention to take the medicine active in their working memory while doing other things [21].

## Materials and Methods

The ethic committee of the Hospital of Padua approved the study. All clinical investigations have been conducted according to the principles expressed in the declaration of Helsinki. All patients gave their written consent before to take part in the study.

A consecutive sample of 207 patients (age: 53.33±13.6; education in years: 8.39±4.19) with end-stage cardiac disease who underwent routine psychological and neuropsychological evaluation prior to acceptance into the heart transplant programme were studied.

None of the patients had previous severe brain injury and or psychiatric illness. Moreover, all patients were assessed by a clinical semi-structured interview and administration of the Minesota Multiphasic Personality Inventory (MMPI-2) [22]. None of them had any abnormal scores in scale of depression (scale D) and in scale of anxiety (scales Pt and A). Patients in our sample were treated with standard medications for chronic compensated heart failure (diuretics, vasodilators, beta-blockers, etc.). This treatment generally improves hemodynamic, thus increasing renal and hepatic function and cerebral flow. During acute phase of congestive heart failure higher dosage of diuretics may cause mental confusion and acute cognitive impairment due to de-hydration or alterations of electrolytes serum levels, but cognitive and psychological assessments were not performed during acutely decompensate heart failure.

Three groups were considered on the basis of the type of cardiac disease: ischaemic dilated cardiomyopathy (IDC); non-ischaemic dilated cardiomyopathy (NIDC); miscellaneous (M). The miscellaneous category included: Cardiomyopathy, hypertrophic cardiomyopathy, congenital heart disease, late post-surgical failure of heart disease. The demographic and clinical characteristics are illustrated in Table 1.

**Table 1:** Clinical features and cardiovascular risk factors by heart disease.

Variables	IDC (n = 75; 39.5%)	NIDC (n = 52; 27.4%)	M (n = 63; 33.2%)	Comparison (p value)*
Sex (males)	93.3%	73.1%	63.5%	p<0.001
Age	2.2±0,5	23.3±6.7	17.1±16.8	ns
Years from diagnosis	29.9%	96.0±2,5	2.3±0,7	p<0.01
Ejection fraction (%)	47.2%	15.6%	96.3±3.5	p<0.001
Cardiac index (L/min/m^2^)	88.3%	36.0%	34.4%	p<0.01
VO_2_max (L/min/Kg)	93.3%	48.8%	17.9%	p<0.01
SaO2 (%)	2.2±0,5	73.1%	34.9%	ns
Mean PAP (mmHg)	29.9%	23.3±6.7	44.1%	ns
Atrial fibrillation	47.2%	96.0±2,5	46.2%	ns
Carotid artery stenosis †	88.3%	15.6%	63.5%	p<0.001
Previous TIA/stroke ‡	93.3%	36.0%	17.1±16.8	ns
Hepatic dysfunction §	2.2±0,5	48.8%	2.3±0,7	ns
Renal dysfunction §	29.9%	73.1%	96.3±3.5	ns
Respiratory dysfunction §	47.2%	23.3±6.7	34.4%	ns
Hyperglycemia ∥	88.3%	96.0±2,5	17.9%	p<0.001
Dyslipidemia	93.3%	15.6%	34.9%	p<0.001
Smokers	2.2±0,5	36.0%	44.1%	p<0.001

IDC: ischemic dilated cardiomyopathy; NIDC: non-ischemic dilated cardiomyopathy; M: miscellaneous cardiac diseases; PAP: pulmonary artery pressure.

*Subgroups were compared by *X* ^2^ (categorical variables) or one-way analysis of variance (continuous variables).

† Medium to high grade.

‡ Transient ischemic attacks (TIA) or stroke.

§ Moderate to severe.

∥ Impaired glucose tolerance (IGF) or impaired fasting glucose (IFG) or diabetes mellitus.

### Neuropsychological measures

All patients underwent routine neuropsychological assessment by the Mini Mental State Examination (MMSE) [23] and by a well-validated battery for the Italian population, the Esame Neuropsicologico Breve (ENB, Short Neuropsychological Examination) [24]. Each patient was assessed individually by experienced neuropsychologists and the assessment test lasted one hour.

The MMSE consists of a brief 30-point questionnaire that is used to screen for cognitive decline (cut-off score: 24). Furthermore, each subject was assessed by the Token test [25]–[26] to verify comprehension. This test assesses verbal comprehension of verbal commands of increasing complexity. The test required patients to point tokens on the basis of their colour, shape and size. The ENB battery investigates different cognitive domains and encompasses thirteen tests: Token test, Trial Making Test, Digit span, Logical story, Interference memory, Cognitive estimation, Abstract verbal reasoning, Phonemic fluency test, Clock drawing test, Overlapping pictures test, Spontaneous drawing, Copy drawing, Ideative and ideomotor praxis test. The tests were chosen to cover five cognitive domains: attention, memory, executive functions, and perceptive and praxis abilities. The cognitive domain of attention included the Trial Making Test A and the Trial Making Test B; the domain of memory included Digit Span, Logical Story and Interference memory tests; the cognitive domain of executive function included Trial Making Test B, Cognitive estimation, Abstract reasoning, Phonemic fluency, Clock drawing, and Overlapping pictures tests. The domain of perception included Spontaneous drawing and Copy drawing tests. One test accounted for more than one domain (Table 2): the Trail Making Test B (TMTB) is a well-known instrument for describing the attentive function but at the same time it evaluates switching ability and working memory (i.e. executive functions); thus, it requires the involvement of executive functions [27].

**Table 2:** Neuropsychological domain and psychometric tests.

Cognitive Domain	Psychometric test	Z mean ± sd	Patients with altered test
Attention	TMT-A	0.25±1.69	12.6%
	TMT-B	0.62±1.83	18.8%
Memory	Digit Span	−0.45±1.11	15.9%
	Logical story: immediate recall	−0.39±1.13	13.5%
	Logical story: delayed recall	−0.62±0.99	15.5%
	Interference memory test 10 s.	−0.45±1.30	21.7%
	Interference memory test 30 s.	−0.62±1.60	19.8%
Executive function	TMT-B	0.62±1.83	18.8%
	Cognitive estimation	−0.90±2.22	27.1%
	Abstract verbal reasoning	−0.22±1.11	11.5%
	Phonemic fluency test	−1.00±1.84	38.2%
	Clock drawing test	−0.92±1.95	23.7%
	Overlapping pictures test	−1.44±1.29	44.4%
Perception	Spontaneous drawing	0.80±2.19	19.8%
	Copy drawing	0.41±1.56	20.8%
Praxis ability	Ideative and ideomotor praxis test	−0.67±2.30	15.0%

Mean, standard deviation and % of patients with deficits (Z scores below 2 standard deviations).

Psychometric tests were expressed by age and education adjusted Z scores, i.e. in units of standard deviation stratified on the basis of age and education level in the reference population of normal individuals. For each test, a Z score equal to or lower than −2 was considered to be abnormal. For each individual, we calculated both the *number of abnormal tests* and the *mean Z psychometric index* (mZPSI) that summarized the Z scores of all tests separately for each cognitive domain. This measure was used as an overall synthetic index of cognitive performance.

A series of statistical analyses was conducted to determine which clinical features and cardiovascular risk factors were significant predictors of cognitive performance. All analyses were performed with SPSS 15.0 (Statistical Package for Social Science, Chicago, IL). All p values were two-tailed and a level of p<0.05 was accepted as statistically significant.

In the first step, data were explored by correlational analysis on continuous clinical variables and scores of cognitive tests (mZPSI for each cognitive domain). In the second step, univariate ANOVA and independent-sample t tests were performed in order to compare cognitive performance, expressed as mZPSI for each cognitive domain on the basis of the category of heart disease, years from diagnosis and different indexes including cardiopulmonary assessment, comorbidities and cardiovascular risk factors. For these analyses, continuous variables for comprehensive pre-transplant cardiopulmonary assessment were considered as categorical cut-off values, used to determine the classification of patients: LVEF = 30%; cardiac index = 2.5 L/min/m2; mean pulmonary artery pressure = 20 mmHg; arterial haemoglobin saturation = 97% (median value); VO2max = 11.2 ml/min/kg (median value); disease duration (years from diagnosis of heat disease) = 6 years (median value). All tests were corrected for multiple comparisons using the false discovery rate method of Benjamini and Hochberg (FDR_BH_) [28]. With this method, tests were corrected for the number of comparisons conducted on a single dependent variable.

### Neurophysiological measures

A subgroup of 60 consecutive patients underwent digital electroencephalographic recording. To evaluate whether this subgroup of patients was comparable to the overall sample we compared patients with and without EEG measures on all the cardiovascular variables (i.e., IC, LVEF, VO2max, etc.) and comorbidities and risk factors (i.e., previous ischemic attach and hepatic dysfunction, etc.) considered in this study. No differences were detected between the two subgroups of patients.

Spontaneous closed-eyes activity was recorded by digital EEG equipment (Brainquick 3200, Micromed, Italy). A standard 21-channel cap (Micromed, Italy) was used, and the electrodes placed according to the 10–20 International System [29]. The EEG tracing was assessed by spectral analysis after visual inspection to exclude artefacts. Spectral analysis was carried out on the derivation T4-O2, T3-O1 and P3-P4 in the frequency range of 1–25.5 Hz [30]. The variables considered for statistical analyses were the mean dominant frequency (MDF), i.e., the mean frequency weighted by the power of each frequency band, and the relative power of alpha, theta, and delta bands, i.e., the percentage of the absolute power of the spectrum due to alpha, theta, and delta activity, respectively. These variables were correlated both with clinical features/cardiovascular risk factors and scores of cognitive performance (mZPSI for each cognitive domain).

## Results

### Neuropsychological results

Results showed that 96% of the patients had a normal ability of comprehension, which is consistent with the high reliability of cognitive tests.

The total sample mean score of the MMSE was 26.9±3.1. Following the norms published by Crums for mean age of 53 years [31], 30% of patients showed impaired MMSE scores, but 86% had one or more cognitive tests altered. The fact that no more than 30% of patients showed impaired MMSE is not surprising since the MMSE may detect cognitive impairment less effectively because of the ceiling effect, the lack of learning condition, and the absence of specific items evaluating executive functions. In fact, detailed neuropsychological evaluation showed that 36% failed five or more tests, indicating broad neuropsychological impairment. In detail, executive functions were impaired in 70%, perception in 31%, memory in 51%, attention in 26%, and praxis abilities in 15% out of the whole sample (Table 2). Cognitive performance in the domain of attention was directly correlated with LVEF (R = 0.17, p<0.05) and inversely correlated with index of renal dysfunction (R = −0.16, p<0.05). Moreover, the global cognitive performance of patients (mZPSI averaged across all cognitive domains) was directly correlated with the cardiac index (R = 0.20, p<0.05) and inversely correlated with index of renal dysfunction (R = −0.17, p<0.05).

A worse performance in the domain of perception was found in patients with a cardiac index <2.5 l/min/m^2^. No significant differences were found in cognitive performance on the basis of pre-transplant VO2max, LVEF, atrial fibrillation/flutter, arterial haemoglobin saturation, or pulmonary artery pressure during right heart catheterization. Regarding comorbidities, previous cerebral ischaemic attacks were associated with significantly lower performances in the memory domain. The presence of hepatic dysfunction was associated with lower performance in the cognitive domain of attention. Regarding atherosclerotic risk factors, hyperglycaemia was associated with lower performance in attention. Detailed results are shown in table 3.

**Table 3:** Clinical features and cognitive performance.

Clinical feature		Cognitive domain			
		perception	memory	attention	Comparison (p value)
Cardiac index (l/min/m^2^)	<2.5	−1.08±1.34			p<0.05
	>2.5	−0.44±1.09			
Previous cerebral ischaemic attacks	yes		−0.96±1.37		p<0.05
	no		−0.49±0.81		
Hepatic dysfunction	yes			−0.94±1.74	p<0.05
	no			−0.05±1.37	
Hyperglycemia	yes			−0.75±1.85	p<0.05
	no			−0.04±1.09	

Values represent results of ANOVA test on mZPSI (see Materials and Methods).

Consistently with previous studies [2], [11], no differences were found in cognitive performance between the aetiological groups considered: ischaemic dilated cardiomyopathy (IDC); non-ischaemic dilated cardiomyopathy (NIDC); miscellaneous (M).

### Neurophysiological results

The ejection fraction of the left ventricle showed an inverse relationship with the electroencephalogram delta relative power on both temporal derivations (R = −0.30 and R = −0.25 on the left and right hemispheres, respectively: both p<0.05) and biparietal derivations (R = −0.27 p<0.05). In addition, the cardiac index was found to be inversely correlated with the electroencephalogram theta relative power in temporal derivations (R = −043, p<0.05 and R = −0.31, p<0.05 on the left and right hemispheres, respectively). The electroencephalogram was also found to be related to the indices of liver function: delta power was directly related to the bilirubin plasma level on both temporal and biparietal derivations (left temporal: R = 0.37, p<0.05; right temporal: R = 0.26, p<0.05; biparietal: R = 0.36, p<0.05) and theta relative power was directly related to γGT (left temporal: R = 0.28, p<0.03; right temporal: R = 0.25, p<0.05 and biparietal: R = 0.30, p<0.05). Cognitive performance in the domain of attention was directly related to theta relative power on both temporal (R = 0.29 and R = 0.33 on the left and right hemispheres, respectively: both p<0.05) and biparietal derivations (R = 0.30, p<0.05).

## Discussion

Increased prevalence (53–58%) of mild cognitive impairment, particularly memory, has previously been demonstrated in older populations with heart failure [3]–[5], [32]. Cognitive dysfunction in these patients correlates with reduced ventricular function; both imaging and neurological examinations have evidenced white matter lesions in older demented patients with heart failure, systolic hypotension, decreased cerebral blood flow and multiple vascular risk factors, as are commonly found in the elderly.

For a long time, during the evaluation of younger candidates for heart transplant, our attention has been focused on psychological findings, assuming that abnormalities in behaviour and the prevalence of depression and anxiety were mainly the result of the seriousness and duration of heart disease. Conversely, the growing amount of data about cognitive deficits after transplantation support the hypothesis of structural cerebral damage related to the status of chronic heart failure. In fact, various causes may be associated with cerebral damage in patients with heart failure, also at a younger age. The combination of abnormal intracardiac blood flow, endothelial dysfunction and rheological abnormalities present in patients with chronic heart failure satisfies Virchow's triad and suggests that, particularly in the setting of left ventricular dysfunction, heart failure is a hypercoagulable state: a risk factor for subclinical or clinical stroke. Moreover, other causes of cerebral damage can include reduced auto-regulation of cerebral circulation, altered cerebrovascular reactivity, increased blood viscosity, and chronic use of drugs (digoxin and over-diuresis). Consequently, it has been suggested that neuropsychological and neurophysiological assessment must be associated with the psychological examination of candidates of heart transplantation, with care being taken to study their executive functioning, which is particularly involved in the pre-transplant decision-making process in terms of the understanding and acceptance of this exceptional event. Moreover, these aspects are determinants for quality of life and the persistence of adherence to treatment after transplant. The prognostic value of cognitive and psychological abnormalities is not exclusively correlated with the severity and duration of cardiopathy, but also with the presence and gravity of morbidity, including cerebral and visceral dysfunctioning.

The results of the present study indicated that end-stage heart failure patients showed a significant decline in cognitive functioning. In accordance with previous studies [11], [12], [5], we found that 86% of our sample obtained abnormal scores in at least one test and that 36% performed within the impaired range in five or more tests, indicating poor performance across a broad range of cognitive domains. These abnormalities were not related to the aetiology of heart disease, but rather to cerebral damage secondary to altered cardiac pump functioning. Even if atherosclerotic risk factors such as diabetes, arterial hypertension, a high plasma level of cholesterol and smoking were more prevalent in patients with IDC, these patients did not show greater cognitive alterations than patients in the other two groups.

We studied the impairments in cognitive functions through an exhaustive neuropsychological investigation in order to delineate the cognitive profile of end-stage heart failure patients. The results showed that the most impaired cognitive domains were those related to executive functions, followed by perception, memory, attention and praxis abilities. Cognitive functions, and particularly executive functions and prospective memory and working memory, are certainly key determinants of an end-stage heart failure transplant recipient's ability to manage the complex treatment regimen and gain maximum benefit from this surgical procedure. Compliance after cardiac transplantation is multifaceted and involves adhering to a prescribed diet and lifestyle, keeping scheduled medical appointments, the maintenance of communication, and, ultimately, adherence to the immunosuppressive regimen.

Our data, in agreement with previous studies [2], [11], [15], corroborated the suspicion that cognitive deficits are unrelated to the aetiology of heart disease. The results showed that other important variables help to delineate the cognitive profile of end-stage heart patients. A low cardiac index (<2.5 L/min/m^2^) was associated with the level of cognitive performance in the domain of perception. Our results also stress the importance of comorbidity; in fact, patients with previous cerebral ischaemic illness showed deficits in the cognitive memory domain. Interestingly, the presence of hepatic dysfunction was associated with deficits in the domain of attention.

Patients estimated to be at higher risk of cognitive deficits may require more attention and more intensive monitoring because these deficits could potentially interfere not just with daily living activities but also with medical adherence. Our data also provided evidence that cognitive deficits are strictly correlated with cerebral hypoperfusion and/or cerebral alteration, as supported by the neurophysiological results. The inverse correlation between the LVEF and electroencephalogram delta power is in agreement with the noxious effect of cerebral hypoperfusion, owing to reduced heart functioning. In fact, an increase in electroencephalogram delta activity is a marker of brain damage [33]. A further index of the harmful effect of hypoperfusion on the brain was provided by the inverse correlation between the cardiac index and electroencephalogram theta power in the temporal derivations. In addition, temporal lobes are known to be highly susceptible to brain hypoperfusion and there is some evidence that mild cognitive impairment could be associated with hypoperfusion in these cortical areas [34]. The relationship between high plasma levels of bilirubin and γGT and a slowing of the electroencephalogram could suggest the possibility of an altered cerebral physiology in patients with secondary liver dysfunction due to heart failure.

Our data on neuropsychological and neurophysiological deficits in end-stage heart disease candidates for transplant stressed the importance of close collaboration across a range of disciplines in order to establish a programme of research and professional education. We need to develop clinical practice guidelines and pathways, which support the implementation of best practice in the assessment and management of comorbid cognitive and neurophysiological alterations in patients with end-stage heart failure.
